# Application of oxycodone in multimodal analgesia for thoracoscopic lung surgery: A single-center retrospective observational study

**DOI:** 10.1097/MD.0000000000047418

**Published:** 2026-01-30

**Authors:** Ran Zhang, Yue Fu, Weixin Zhang, Xiaoyi Wang

**Affiliations:** a Department of Anesthesiology, Peking University People’s Hospital, Beijing, China; b Department of Anesthesia, Tibet Autonomous Region People’s Hospital, Lhasa, China.

**Keywords:** multimodal analgesia, oxycodone, postoperative analgesia, thoracoscopic surgery

## Abstract

In video-assisted thoracoscopic surgery, sufentanil patient-controlled intravenous analgesia (PCIA) demands continuous infusion, raising opioid load and side-effects while lacking κ-receptor visceral coverage. Oxycodone’s dual µ/κ profile and longer action allow demand-only PCIA. We compared the 2 within a standardized TPVB-NSAID protocol. Single-center retrospective study (Jan 2017–Dec 2018). Adults undergoing video-assisted thoracoscopic lung surgery with multimodal analgesia were allocated to sufentanil (S) or oxycodone (O) PCIA. After 1:1 propensity matching (n = 527 per group), opioid consumption, numerical rating scale pain, analgesic demands, PONV, dizziness, sleep quality and length of stay were compared. Matched groups were comparable demographically and intraoperatively. Postoperative days 1 to 2 opioid use (morphine equivalents) was lower in O (0.8 mg and 1.2 mg) than S (27.4 mg and 36.5 mg; *P* < .001). Resting numerical rating scale on day 2 favored O (*P* = .043); exercise scores were similar. Patients pressed the O pump more often (2 vs 0 and 3 vs 1; *P* <.01). PONV day-1: O 1.5 %, S 6.5 % (*P* <.001); dizziness: 3.8 % vs 9.9 % (*P* <.001). Good sleep reported in 73.1 % O vs 61.9 % S (*P* <.001). Postoperative stay shorter in O (*P* = .002). Within a TPVB-NSAID multimodal regimen, oxycodone-based PCIA provided equivalent analgesia, lower opioid consumption and fewer adverse effects than sufentanil-based PCIA, and was associated with earlier discharge after thoracoscopic lung surgery.

## 1. Introduction

Postoperative pain is a critical determinant of recovery after thoracic surgery.^[1,2]^ The advent of minimally invasive techniques, particularly video-assisted thoracoscopic surgery (VATS), has markedly reduced incisional trauma and shortened hospital stay compared with open thoracotomy^[3]^; nevertheless, adequate control of acute postoperative pain remains elusive for many patients undergoing thoracoscopic lung resection.^[4]^

Multimodal analgesia – combining regional anesthesia, nonsteroidal anti-inflammatory drugs (NSAIDs) and opioids – is currently the most effective strategy for managing this pain.^[5,6]^ Thoracic paravertebral block (TPVB) provides excellent early analgesia,^[7]^ but its duration is limited; NSAIDs target inflammatory pain yet offer only modest analgesic potency. Consequently, opioids are still required as the principal analgesic component.

Opioid selection profoundly influences both analgesic quality and adverse-event profile.^[8]^ Sufentanil, a highly selective µ-opioid receptor agonist, is widely used in China for postoperative patient-controlled intravenous analgesia (PCIA) because of its rapid onset and high potency.^[9,10]^ Its short context-sensitive half-life, however, mandates a continuous background infusion to maintain analgesia, increasing total opioid exposure and the incidence of nausea, vomiting, dizziness and respiratory depression. Moreover, sufentanil lacks clinically relevant κ-receptor activity, limiting its efficacy against the visceral and central sensitization components of thoracoscopic pain.

Oxycodone is a semisynthetic opioid that acts as a dual agonist at both µ- and κ-opioid receptors.^[11]^ This receptor profile provides broader analgesic coverage, including visceral pain, and a longer duration of action, allowing effective analgesia with a demand-only PCIA regimen and no background infusion. These pharmacological advantages translate into lower cumulative opioid consumption and potentially fewer dose-related side-effects.

We therefore hypothesized that, within a standardized multimodal protocol incorporating TPVB and NSAIDs, oxycodone-based PCIA would provide analgesia equivalent or superior to sufentanil-based PCIA while reducing opioid use and adverse events. This retrospective observational study compares analgesic efficacy, opioid consumption, side-effects and recovery outcomes between oxycodone- and sufentanil-based PCIA in adults undergoing thoracoscopic lung surgery.

## 2. Materials and methods

### 2.1. Study design

This is a single-center retrospective observational study using propensity-score matching (PSM) to investigate whether the use of oxycodone in multimodal analgesia after thoracoscopic lung surgery has advantages over sufentanil. The study protocol was reviewed and approved by the Academic Committee of Peking University People’s Hospital (approval number: 2020PHB308-01). As this study is a retrospective study, the ethics committee believes that obtaining informed consent is not necessary, thus eliminating the requirement for obtaining informed consent. Patient data are strictly confidential.

### 2.2. Data collection

Data from all patients aged 18 years or older who underwent thoracoscopic lung surgery at Peking University People’s Hospital from January 2017 to December 2018 were obtained from the electronic medical record system. Patients who did not receive preoperative TPVB, open or intermediate open thoracotomy, emergency surgery, and postoperative admission to the intensive care unit were excluded from this study. Patients who did not receive postoperative PCIA, patients with contraindications to NSAIDs medication and patients who did not receive analgesic follow-up on postoperative days 1 and 2 were also excluded. According to the postoperative analgesic regimen, they were divided into a sufentanil-containing multimodal analgesia group (Group S) and an oxycodone-containing multimodal analgesia group (Group O).

A research assistant who does not participate in data analysis will use the electronic medical record system and analgesic follow-up system of Peking University People’s Hospital to review past medical records, screen patients who meet the inclusion criteria, and collect relevant information about the cases. The collected data include: demographic indicators (age, gender, height, weight), previous diseases (hypertension, diabetes, coronary heart disease, pulmonary disease), intraoperative data (intraoperative sufentanil dosage, remifentanil dosage), Postoperative data (opioid dosage 1 to 2 days postoperatively, NRS rest and exercise scores 1 to 2 days postoperatively, number of analgesic pump presses, incidence of nausea and vomiting, dizziness, sleep status, and postoperative hospital stay).

All postoperative opioids were counted, and for statistical comparisons, opioids were converted to morphine equivalent units in the following ratio: sufentanil: fentanyl: oxycodone: morphine = 1:10:1000:1000 [8].

### 2.3. Anesthesia method

Anaesthesia was induced using propofol 1.5 to 2 mg.kg^−1^ or etomidate 0.2 to 0.3 mg.kg^−1^, cisatracurium 0.3 to 0.4 mg.kg^−1^ or rocuronium 0.5 to 0.6 mg.kg^−1^, and sufentanil 0.2 to 0.4 μg.kg^−1^ and/or remifentanil 0.5 to 2 μg.kg^−1^. The maintenance of anesthesia used propofol and remifentanil. The propofol dosage was adjusted during the procedure to maintain the bispectral index (BIS) between 40 and 60, and muscle relaxants were applied according to the muscle relaxation interval. The remifentanil dose was adjusted to maintain blood pressure and heart rate between 80% and 120% of basal blood pressure and heart rate. At the same time, additional sufentanil was administered in appropriate doses according to the changes in the heart rate and blood pressure during surgery. Before the end of the procedure, all patients were intravenously administered 8 mg of ondansetron or 5 mg of tropisetron.

### 2.4. Pain management

Patients were treated with a thoracic paravertebral block before thoracoscopic surgery followed by double-lumen tracheal intubation under general anesthesia.

Paravertebral block was conducted under off-plane ultrasound guidance with a high-frequency (4–8 MHz) ultrasound transducer probe (GE Logiq E, Boston, USA). The probe was positioned vertically in a sagittal, paramedian plane, parallel to the spinous process, at the predetermined level, and the paravertebral space (PVS) was identified. After infiltrating the skin with local anesthetic (2% lidocaine), a 22-gauge Quincke tip spinal needle was advanced into the PVS under ultrasound guidance, using an off-plane technique. Once the tip of the needle was positioned between the superior costotransverse ligament and the pleura, and after negative aspiration for blood or air, 2 mL of sterile 0.9% saline was injected to confirm the anterior displacement of the parietal pleura. A total of 15 mL of 0.5% ropivacaine was then deposited in the thoracic paravertebral space at T3 and again at T6 (30 mL in total), with real-time ultrasound verification of the solution spread.

Group S received postoperative analgesia via a patient-controlled analgesia (PCA) pump containing sufentanil 250 µg diluted to 250 mL with 0.9 % saline. The pump was programmed with a background infusion of 1.5 to 3 mL h^−1^, a demand bolus of 2 to 3 mL, a 15-minutes lockout, and an hourly maximum of 15 mL.

Group O was managed with an oxycodone PCA pump prepared by dissolving 40 mg of oxycodone in 0.9 % saline to a total volume of 100 mL. Settings were: no basal infusion, a 2.5 mL bolus, a 5-min lockout, and an hourly limit of 20 mL.

All patients received flurbiprofen axetil 100 mg/12 hours after surgery. In accordance with the standard postoperative pain-management protocol of our hospital, whenever the postoperative numerical rating scale (NRS) exceeded 3, analgesic therapy was initiated via the PCA pump. If pain had not eased after 1 hour, rescue consisted of either oral Tylenol (oxycodone 5 mg combined with acetaminophen 375 mg) or, alternatively, an intramuscular injection of fentanyl 0.05 mg or morphine 5 mg.

### 2.5. Observation indicators

The primary observation indicators were the incidence of adverse reactions, including nausea, vomiting, and dizziness, as well as sleep conditions on postoperative days 1 and 2. Secondary observation indicators included opioid consumption and pain scores on postoperative days 1 and 2, and the length of hospital stay postsurgery.

The reason for selecting data from 1 to 2 days after surgery is that the acute pain management period typically spans 1 to 2 days postsurgery. Additionally, most patients have had their chest tubes removed within 3 days postsurgery, by which time pain has significantly diminished and many patients have been discharged.

The definition of postoperative nausea and vomiting is nausea and vomiting grade ≧1 (nausea and vomiting grading, grade 0 is no nausea or vomiting; grade 1 is mild nausea without vomiting; grade 2 is mild nausea with vomiting 1 to 2 times/day; grade 3 is moderate nausea with vomiting 3 to 5 times/day; grade 4 is severe nausea with vomiting >5 times/day).

The pain score was assessed using the numeric rating scales (NRS) for both rest and movement. All scores were assessed by specially trained anesthesiology nurses.

At our institution, the assessment of postoperative sleep quality is primarily conducted through regular follow-ups by nurses and documented on the postoperative pain follow-up form. The evaluation of sleep quality is recorded as either good or poor.

### 2.6. Statistical analysis

Continuous variables are presented as mean ± SD or median (25th–75th percentile) with 95% CI for the difference between groups; categorical variables are reported as % (95% CI). Inter-group comparisons were performed with Student *t* test, Mann–Whitney *U* test or χ² test as appropriate. A 2-sided *P* <.05 was considered significant. All analyses were performed with SPSS 25.0 and GraphPad Prism 8.

In order to eliminate confounding factors, we used a 1:1 PSM to match the 2 groups of data. A variable of *P* <.2 was chosen for the matching factor, and a matching tolerance of 0.02 was chosen. The 2 groups were then compared for nausea and vomiting, dizziness, and sleep, as well as for postoperative NRS scores and postoperative hospitalization days. *P* <.05 was considered statistically significant. All tests were 2-sided tests.

## 3. Results

**Figure 1.**
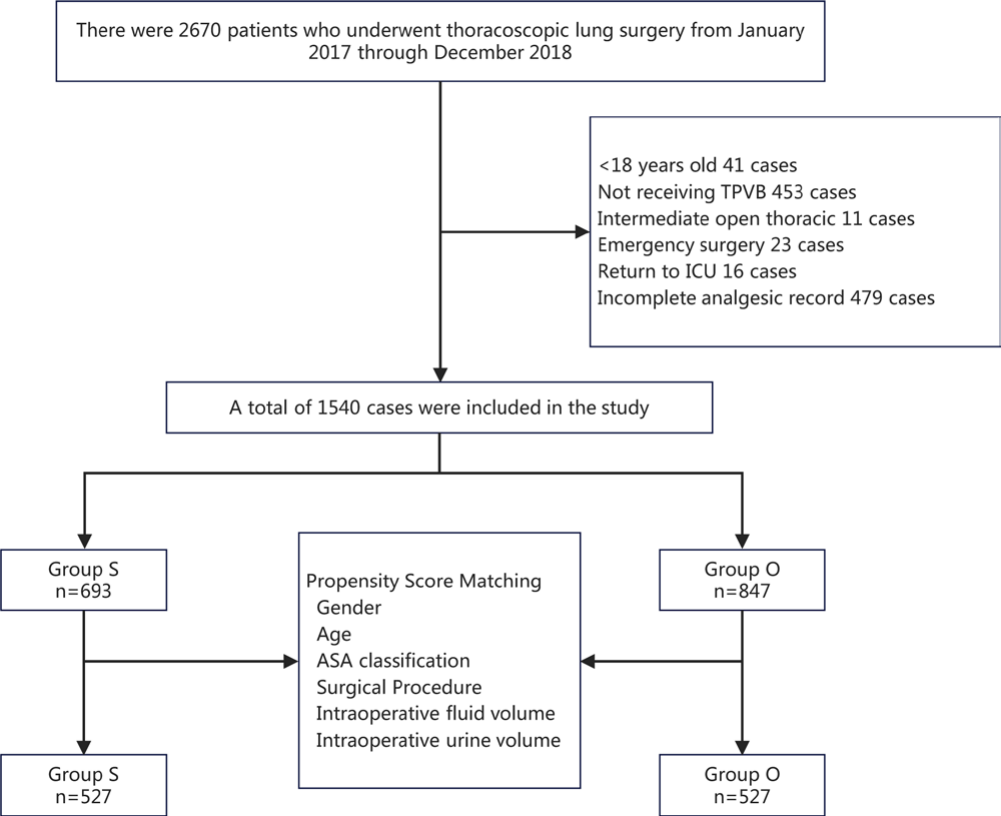
Flow chart of the study. ASA = American Society of Anesthesiologists, Group O = oxycodone-containing multimodal analgesia group, Group S = sufentanil-containing multimodal analgesia group, ICU = intensive care unit, TPVB = thoracic paravertebral block.

From January 2017 to December 2018, a total of 2670 patients underwent thoracoscopic lung surgery at Peking University People’s Hospital, including 41 patients aged <18 years, 453 patients who did not receive thoracic paravertebral block, 11 cases were converted to thoracotomy, 23 patients who were emergent surgery, 16 patients who were transferred to ICU for further treatment after surgery, 107 patients with NSAIDs drug application contraindications, and 479 patients with incomplete postoperative analgesia records. Finally, a total of 1540 cases were included in the study, of which 693 patients received multimodal analgesia regimen including sufentanil (group S), and 847 patients received multimodal analgesia regimen including oxycodone (group O) (Fig. 1).

### 3.1. Comparison of the first 2 groups of PSM

There were significant differences in ASA classification, intraoperative fluid infusion volume, and intraoperative urine output between the 2 groups (*P* <.05). There were no significant differences in gender composition, age, and surgical type between the 2 groups. There were no statistical differences in BMI, preoperative comorbidities, operation time, intraoperative bleeding volume, intraoperative sufentanil dose, and intraoperative remifentanil dose between the 2 groups (*P* >.05) (Table 1).

**Table 1:** Comparison results of the 2 groups before PSM.

	Group S (n = 693)	Group O (n = 847)	*P*	95% CI
Gender (male/female), n	286/407	384/463	.121	0.847 (0.692–1.038)
Age (year)	58 ± 11	59 ± 11	.191	−0.067 (−0.167 to 0.033)
BMI (kg/m^2^), mean ± SD	24.1 ± 3.3	24.1 ± 3.1	.938	−0.004 (−0.104 to 0.096)
ASA (1/2/3), n	303/354/36	127/671/49	<.001	–
Preoperative comorbidities, n (%)				
Hypertension	221 (31.9)	269 (31.8)	1.000	0.994 (0.801–1.233)
Diabetes	87 (12.6)	108 (12.8)	.939	1.018 (0.753–1.377)
Coronary heart disease	58 (8.4)	57 (6.7)	.243	0.790 (0.540–1.155)
Pulmonary complications	16 (2.3)	15 (1.8)	.471	0.763 (0.374–1.554)
Surgical classification, n			.132	1.178 (0.954–1.454)
Pleuropneumonectomy (including segmentectomy)	254	279	–	–
Lobectomy (including sleeve resection)	439	568	–	–
Surgery time (min), mean ± SD	118 ± 55	121 ± 52	.276	−0.056 (−0.156 to 0.045)
Intraoperative bleeding volume (mL), median (IQR)	30 (20, 50)	30 (20, 50)	.309	–
Intraoperative infusion volume (mL), mean ± SD	1368 ± 415	1309 ± 411	.005	0.144 (0.044–0.245)
Urine volume during the operation (mL), median (IQR)	500 (250, 800)	400 (200, 700)	<.001	–
Sufentanil consumption (µg), mean ± SD	28.2 ± 7.3	28.3 ± 6.6	.849	−0.010 (−0.110 to 0.091)
Remifentanil consumption (µg/kg/min), mean ± SD	0.09 ± 0.04	0.09 ± 0.04	.742	−0.024 (−0.124 to 0.077)

ASA = American Society of Anesthesiologists, BMI = body mass index, CI = confidence interval, Group O = oxycodone analgesia group, Group S = sufentanil analgesia group, IQR = inter-quartile range, PSM = propensity-score matching, SD = standard deviation.

### 3.2. Comparison between the 2 groups after PSM

There was no significant difference in various indicators between the 2 groups after PSM (Table 2).

**Table 2:** Comparison results between the 2 groups after PSM

	Group S (n = 527)	Group O (n = 527)	*P*-value	95% CI
Gender (male/female), n	224/303	223/304	1.000	1.008 (0.789–1.287)
Age (year), mean ± SD	58 ± 11	58 ± 11	.960	0.003 (−0.118 to 0.124)
BMI (kg/m^2^), mean ± SD	24.1 ± 3.4	24.1 ± 3.1	.918	0.006 (−0.114 to 0.127)
ASA (1/2/3), n	149/342/36	127/372/28	.133	–
Preoperative comorbidities, n (%)				
Hypertension	172 (32.6)	172 (32.6)	1.000	1.000 (0.773–1.294)
Diabetes	67 (12.7)	60 (11.4)	.57	0.882 (0.608–1.279)
Coronary heart disease	50 (9.5)	34 (6.5)	.087	0.658 (0.418–1.035)
Pulmonary complications	14 (2.7)	8 (1.5)	.281	0.565 (0.235–1.358)
Surgical classification, n				
Pleuropneumonectomy (including segmentectomy)	184	343	.748	0.951 (0.739–1.225)
Lobectomy (including lobectomy with sleeve resection)	190	337		
Surgery time (min), mean ± SD	116 ± 52	122 ± 53	.070	−0.112 (−0.233 to 0.009)
Intraoperative bleeding volume (mL), median (IQR)	30 (20, 50)	30 (20, 50)	.099	0.000 (0.000–0.000)
Intraoperative infusion volume (mL), mean ± SD	1327 ± 404	1335 ± 423	.757	−0.019 (−0.140 to 0.102)
Urine volume during the operation (mL), median (IQR)	450 (200, 700)	500 (200, 800)	.658	0.000 (−50.000 to 0.000)
Sufentanil consumption (µg), mean ± SD	28.2 ± 7.2	28.4 ± 6.6	.727	−0.022 (−0.142 to 0.099)
Remifentanil consumption(µg/kg/min), mean ± SD	0.09 ± 0.04	0.09 ± 0.04	.405	−0.051 (−0.172 to 0.069)

ASA = American Society of Anesthesiologists, BMI = body mass index, CI = confidence interval, Group O = oxycodone analgesia group; Group S = sufentanil analgesia group; PSM = propensity-score matching, SD = standard deviation.

### 3.3. Postoperative analgesia

On postoperative day 1, resting and dynamic NRS scores did not differ between groups. Opioid consumption was lower in Group O than in Group S (0.8 mg vs 27.4 mg, *P* <.001), whereas the number of PCA demands was higher (2 vs 0, *P* <.01).

On day 2, resting NRS remained lower in Group O (*P* = .043); dynamic scores were similar (*P* = .510). Total opioid use was again lower in Group O (1.2 mg vs 36.5 mg, *P* <.001), with more PCA activations (3 vs 1, *P* <.01).

Rescue analgesia was required by 31 patients in Group S (oral oxycodone/acetaminophen 21, i.m. morphine 10) and 28 in Group O (oral oxycodone/acetaminophen 21, i.m. morphine 10); the difference was not significant (Table 3).

**Table 3:** Comparison of analgesia between the 2 groups.

	Group S (n = 527)	Group O (n = 527)	*P*-value	95% CI
POD 1, median (IQR)				
NRS resting	0 (0, 2)	0 (0, 2)	.469	0.000 (0.000–0.000)
NRS movement	3 (0, 4)	3 (2, 4)	.252	0.000 (0.000–0.000)
MEU (mg)	27.4 (22.6, 34.6)	0.8 (0.0, 1.6)	<.001	26.174 (25.425–26.939)
Number of analgesia pump presses (times)	0 (0, 2)	2 (0, 4)	<.001	−1.000 (−1.000 to 0.000)
POD 2, median (IQR)				
NRS resting	0 (0, 1)	0 (0, 1)	.043	0.000 (0.000–0.000)
NRS movement	3 (0, 3)	2 (1, 3)	.51	0.000 (0.000–0.000)
MEU (mg)	36.5 (33.2, 47.2)	1.2 (0.4, 2.8)	<.001	34.930 (34.238–35.718)
Number of analgesia pump presses (times)	1 (0, 4)	3 (1, 7)	<.001	−1.000 (−2.000 to −1.000)
Rescue analgesia POD 1				
Morphine, n (%)	10 (1.9)	7 (1.3)	.787	0.898 (0.531–1.519)
Tylenol, n (%)	21 (4.0)	21 (4.0)		
Rescue analgesia POD 2				
Morphine, n (%)	0	0	–	–
Tylenol, n (%)	0	0	–	–

Group O = oxycodone analgesia group; Group S = sufentanil analgesia group; MEU = morphine equivalent dose; NRS = numeric rating scales.

### 3.4. Adverse reactions and postoperative hospitalization time

On the first day after surgery, the incidence of nausea and vomiting in group O was significantly lower than that in group S (1.5% vs 6.5%, *P* <.001). There was no significant difference in the incidence of nausea and vomiting between the 2 groups on the second day after surgery (0.2% vs 1.1%, *P* = .124). The incidence of dizziness in group O was lower than that in group S (3.8% vs 9.9%, *P* <.001). The sleep condition after surgery in group O was better than that in group S (73.1% vs 61.9%, *P* <.001). The hospitalization time after surgery in group O was shorter than that in group S (*P* = .002) (Table 4).

**Table 4:** Comparison of adverse reactions and hospitalization time between the 2 groups.

	Group S (n = 527)	Group O (n = 527)	*P*-value	95% CI
Postoperative nausea and vomiting, n (%)				
POD 1	34 (6.5)	8 (1.5)	<0.001	0.224 (0.102–0.488)
POD 2	6 (1.1)	1 (0.2)	0.124	0.165 (0.020–1.376)
Dizziness, n (%)	52 (9.9)	20 (3.8)	<0.001	0.360 (0.212–0.613)
Sleep condition (sleep well), n (%)	326 (61.9)	385 (73.1)	<0.001	0.598 (0.461–0.776)
Postoperative hospitalization days, median (IQR)	4 [4,6]	4 [3,5]	0.002	0.000 (0.000–0.000)

Group O = oxycodone analgesia group, Group S = sufentanil analgesia group.

## 4. Discussion

The principal finding of this study is that, within a TPVB-NSAID multimodal regimen, oxycodone-based PCIA provided equivalent analgesia to sufentanil-based PCIA while reducing cumulative opioid consumption by approximately 95 % and lowering the incidence of PONV, dizziness and sleep disturbance, resulting in a 1-day reduction in median hospital stay.

Sufentanil is widely used for postoperative analgesia in China because it is a potent opioid with only modest respiratory-depressant activity.^[9,10]^ Its clinical duration is short, so PCA is almost always supplemented with a low background infusion to reduce the number of demands and improve patient satisfaction. Oxycodone, on the other hand, is a long-acting opioid; a continuous background infusion readily produces drug accumulation and a higher incidence of side-effects, so background dosing is usually omitted. These diverging administration strategies explain most of the between-group differences in total opioid consumption. Nevertheless, even when the same PCA regimen is used, sufentanil consumption remains comparatively high. Wang et al, in a study of laparoscopic gastrectomy without background infusion, reported significantly greater sufentanil than oxycodone use.^[12]^ Because opioid-related adverse events are dose-dependent, the sufentanil group also experienced more nausea, vomiting, dizziness and other side-effects.

Visceral pain is a form of nociceptive pain that originates from the internal organs, typically triggered by mechanical stretching, spasm, ischemia, or inflammation, and often produces deep, poorly localized discomfort.^[13]^ Pre-clinical and clinical data indicate that κ-opioid receptors play a particularly important role in suppressing such pain. Oxycodone is a dual μ- and κ-receptor agonist; its κ-receptor activity confers a specific inhibitory effect on visceral nociceptive input. In a recent abdominal-surgery trial, Han et al reported that patients receiving oxycodone experienced fewer opioid-related adverse events and expressed greater satisfaction than those given sufentanil,^[14]^ findings that mirror the present study. Postoperative distress is not always expressed as overt pain; subtle residual discomfort can still disrupt sleep. By attenuating this visceral discomfort, oxycodone improves overall postoperative comfort and, consequently, sleep quality.

Research limitations: First, the retrospective design precludes causal inference and is susceptible to unmeasured confounders despite PSM. Second, the single-center setting may limit generalisability. Third, outcomes such as sleep quality were recorded as binary variables (good vs poor) rather than with a validated instrument, potentially introducing assessment bias. Finally, we did not collect data on chronic postsurgical pain or long-term opioid use. Large, multi-center randomized trials are warranted to confirm these findings.

In summary, within a TPVB-NSAID multimodal regimen, oxycodone-based, demand-only PCIA delivered equivalent analgesia, markedly lower opioid consumption and fewer adverse effects than sufentanil-based PCIA, and was associated with earlier discharge after thoracoscopic lung surgery. These data support a formulary shift towards κ-active opioids for VATS analgesia and provide the rationale for a large-scale, multi-center randomized trial powered to detect differences in chronic pain and health-economic outcomes.

## Acknowledgments

We thank the anesthesiologists and nurses who participated in intraoperative anesthesia at the Department of Anesthesiology, Peking University People’s Hospital.

We thank Dr Xiaoyan Yan for statistical guidance.

## Author contributions

**Data curation:** Yue Fu, Weixin Zhang, Xiaoyi Wang.

**Formal analysis:** Yue Fu.

**Investigation:** Ran Zhang.

**Methodology:** Ran Zhang.

**Project administration:** Ran Zhang.

**Supervision:** Ran Zhang

**Validation:** Yue Fu.

**Writing – review & editing:** Ran Zhang.
